# Leg pain relief after discography (LPRAD) in lumbar disc herniation: A retrospective analysis of an interesting phenomenon

**DOI:** 10.1097/MD.0000000000047423

**Published:** 2026-01-30

**Authors:** Zhenyuan Lu, Zhongyang Fan, Lu Lin, Yu Du, Yang Wang, Yun Cheng, Zhenyong Ke, Xiaolin Chen

**Affiliations:** a Department of Spine Surgery, The Second Affiliated Hospital of Chongqing Medical University, Chongqing, China.

**Keywords:** discography, leg pain, lumbar disc herniation

## Abstract

In clinical practice, we have observed that certain patients with lumbar disc herniation (LDH) experience leg pain relief after discography (LPRAD). This study aims to investigate and analyze the specific factors contributing to the LPRAD phenomenon. A retrospective analysis was conducted on patients with LDH who underwent discography. Patients were categorized into relief and non-relief groups based on postoperative visual analog scale (VAS) scores for leg pain. Comparisons of demographic, radiographic, and clinical outcomes were performed between the groups. The leg pain VAS was assessed at the 3-month follow-up. Sixty-nine cases were included in the final analysis. Of these, 22 patients who reported a leg pain VAS of less than 3 after discography were designated as the relief group, while the remaining 47 patients constituted the non-relief group. A proportion of patients (22.45%) experienced significant leg pain relief after discography. No significant differences were observed in baseline (*P* > .05). Before discography, the relief group exhibiting a greater disc height (*P* < .05). Additionally, the LDH level range of motion at the LDH level was significantly greater in the relief group (*P* < .05). The superior–inferior dimension of disc protrusions was also significantly smaller in the relief group (*P* < .05). We identified a phenomenon termed LPRAD, observed in 22.45% of our study cohort. This phenomenon may be linked to contrast-induced pressure changes in protruding anatomical regions and the dilution mechanisms of inflammatory mediators, and may help clinicians predict those who could benefit from discography.

## 1. Introduction

Symptomatic lumbar disc herniation (LDH) is widely recognized as the predominant cause of both lower back pain and sciatica, with a prevalence estimated to range between 3% and 5%.^[1,2]^ Discography is a diagnostic technique employed to evaluate intervertebral disc pathology.^[3–5]^ This method involves the direct injection of contrast media into the intervertebral disc, which can reveal structural changes and the extent of pathology within the disc, thereby assisting clinicians in formulating more precise treatment plans.^[6]^ In clinical practice, an intriguing phenomenon has been observed that warrants further investigation: some patients experience significant relief from lower limb pain symptoms following discography. All patients undergoing angiography had not responded to conservative treatment, and part of them experienced immediate relief of lower limb pain after the angiography procedure. This immediate post-procedural relief distinguishes leg pain relief after discography (LPRAD) from the well-documented spontaneous regression of symptoms, which typically occurs over a longer timeframe.^[7]^ This study challenges the conventional view of discography as merely a diagnostic tool.^[4,8]^ Traditionally utilized for the precise identification of intervertebral disc lesions responsible for lower back pain and/or leg pain, our clinical cases suggest that, for certain specific patient populations, discography not only yields valuable diagnostic insights but also unexpectedly exerts a therapeutic effect by reducing pain. Despite discography’s well-documented efficacy in diagnosing low back pain, there is a paucity of research regarding its application in the treatment of lower limb pain. Therefore, this retrospective study aimed to document the incidence of the LPRAD phenomenon, and identify the specific clinical and radiographic factors associated with its occurrence. We hypothesized that patients experiencing LPRAD would exhibit distinct disc morphological characteristics on imaging compared to those who did not.

## 2. Materials and methods

### 2.1. Patient characteristics

A retrospective analysis was conducted on the clinical outcomes of patients clinically suspected of LDH who underwent discography at our institution between January 2019 and December 2024, with all procedures performed by spine surgeons possessing a minimum of 5 years of specialized surgical experience.

*Inclusion criteria included*: Patients with a well-documented history of low back and leg pain, with a clear association between the pain and intervertebral disc herniation; Patients’ MRI and CT findings did not correlate with the clinical symptoms, a discography was required to pinpoint the painful disc level. Patients who successfully underwent discography.

*Exclusion criteria included*: Patients exhibiting severe allergic reactions to contrast agents; Presence of other identifiable sources of pain, such as spinal fractures, tumors, or infections; A history of previous lumbar spine surgery; Occurrence of severe complications during or after discography, such as infections of the intervertebral disc space or nerve injuries; Undergoing therapeutic blocking procedures with steroids during surgery or receiving analgesic medications postoperatively; Incomplete postoperative lumbar CT data.

### 2.2. Surgical technique

All surgical procedures were conducted by the same surgeon possessing of 5 years of specialized surgical experience. Participants in each relief group were positioned in the prone position. All discography procedures were performed via a standard posterior approach. The approach was based on patient characteristics.

Standard surgical preparation was followed, including cleaning and draping the patients with sterile cloths. The skin entry point was determined using fluoroscopy. An 18-gauge needle was utilized to access the intervertebral disc bypass the facet joint process under local anesthesia. Upon reaching the center of the disc, 2 mL of contrast agent (Iohexol, Omnipaque) was administered under fluoroscopic guidance. Throughout the procedure, only superficial anesthesia was administered. The replication of the patient’s pain was recorded. A CT scan was performed within 2 hours post-surgery. The identification of the pain-generating level and the specific surgical level was determined by the induction of intraoperative lower limb pain and the verification of contrast leakage through postoperative CT scans.

### 2.3. Evaluation

Baseline data included age, gender, lower limb pain time, and body mass index.

Radiologic characteristics and outcomes assessed included visual analog scale (VAS) scores for leg pain, the interval between the lumbar CT scan and the conclusion of the surgery, and the results of the pain provocation test. Leg pain VAS was assessed within 2 hours after the discography procedure and the 3-month follow-up. All imaging studies (MRl, CT, and X-ray) were performed and evaluated at our institution. Lumbar disc herniation zones and Pfirrmann classification^[9]^ were evaluated using MRI. The classification of lumbar Dallas discogram description,^[10]^ compression site (shoulder/axillary region), protrusion dimensions (anteroposterior, transverse, and superior–inferior), and the ratio of the anteroposterior diameter of the nerve root at the compression level on the affected side to that on the healthy side were assessed using CT imaging. Disc height, LDH level lumbar range of motion (ROM) were assessed using X-ray.

### 2.4. Statistical analysis

Statistical analyses were conducted using SPSS software (version 23.0, SPSS, IBM Corp, Armonk). Categorical data were summarized using counts and percentages, while continuous variables were summarized using means and standard deviations. The chi-square test was employed for categorical data analysis, and an independent t test was utilized to evaluate clinical outcomes. Statistical significance was determined with 2-sided *P*-values of <.05.

### 2.5. Ethics approval and consent to participate

The study was approved by the Institutional Ethics Committee of the Second Affliated Hospital of Chongqing Medical University, and was conducted according to the ethical standards of Helsinki Declaration (1964) and its subsequent amendments. Informed consent was obtained from all subjects and/or their legal guardians.

## 3. Results

### 3.1. Patient baseline characteristics

Table 1 provides the baseline characteristics of the 2 groups under study. No statistically significant differences were found between the groups in terms of age, gender, body mass index, lower limb pain time, discography level, Pfirrmann grade, LDH region, or compression location (*P* > .05, Table 1). However, disc height was significantly different between the groups, with the relief group exhibiting a mean disc height of 10.92 ± 2.95 mm, compared to 9.62 ± 1.14 mm in the non-relief group. Additionally, the ROM was significantly greater in the relief group, at 7.16 ± 2.92°, compared to 4.72 ± 2.58° in the non-relief group (both *P* < .05, Table 1). Furthermore, the superior–inferior dimension of the protrusions was significantly smaller in the relief group, measuring 5.7 ± 1.5 mm, compared to 6.6 ± 1.6 mm in the non-relief group (*P* < .05, Table 1).

**Table 1:** Baseline and radiologic characteristics of the two groups.

	Non-relief (n = 47)	Relief (n = 22)	*P* value
Age, yr	58.49 (13.28)	61.09 (12.14)	NS
Gender, F/M	23/24	10/12	NS
Lower limb pain time, mo	11.04	8.63	NS
Body mass index, kg/m^2^	25.2 (0.2)	25.0 (0.7)	NS
LDH level			NS
L2/3	0	1	
L3/4	7	1	
L4/5	26	15	
L5/S1	14	5	
Disc height, mm	9.62 (1.14)	10.92 (2.95)	<.05
ROM	4.72 (2.58)	7.16 (2.92)	<.05
LDH region			NS
1	10	3	
2	36	15	
3	1	4	
Pfirrmann			NS
2	1	0	
3	21	11	
4	25	11	
Compression location			NS
Shoulder	26	15	
Axilla	21	7	
Protrusions, mm			
Anterioposterior diameters	3.5 (1.1)	3.1 (0.9)	NS
Transverse diameters	7.2 (2.1)	6.5 (2.6)	NS
Superior–inferior diameters	6.6 (1.6)	5.7 (1.5)	<.05
Nerve roots ratio	0.64 (0.11)	0.69 (0.14)	NS

Values are presented as mean (SD) except for M:F ratio.

F = female, M = male, NS = not significant, VAS = visual analog scale.

### 3.2. Clinical and radiological outcome

**Figure 1.**
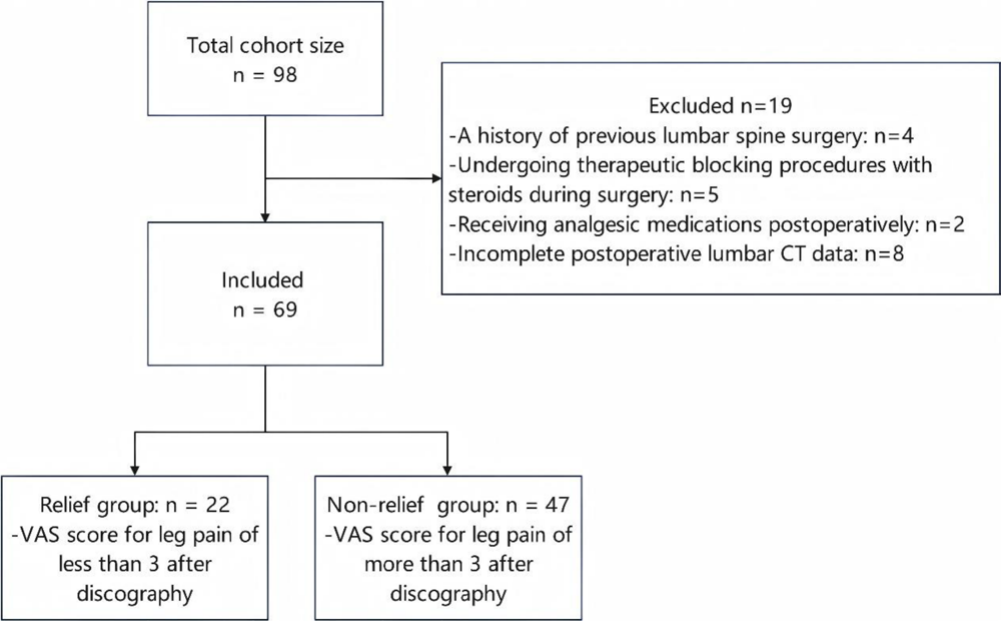
Study population.

Between January 2019 and December 2024, a total of 69 patients meeting the selection criteria were enrolled in the study, of these, 22 patients, who reported a VAS score for leg pain of less than 3 after discography, were classified as the relief group. The remaining 47 patients were categorized as the non-relief group. The analysis was conducted on 69 patients. However, to provide a conservative estimate, the incidence of lower limb pain relief 22.45% (22 out of 98) was calculated using all 98 initially identified patients, including the 19 excluded for incomplete follow-up (Fig. 1). Patients in the relief group were discharged without surgery due to alleviation of lower limb pain, while all patients in the non-relief group underwent endoscopic surgical treatment.

**Figure 2.**
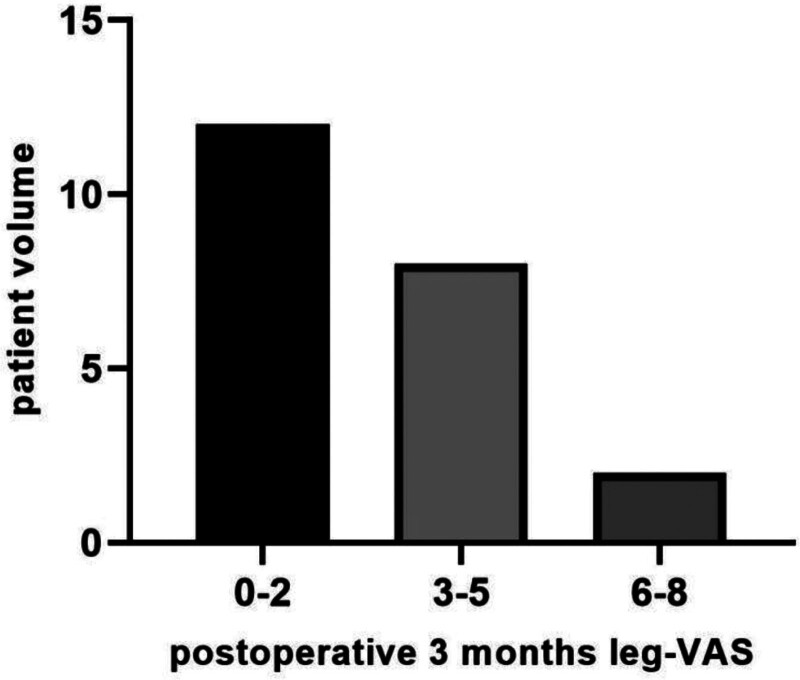
The relief group with 3-months postoperative VAS follow-up data for lower limb pain.

In the relief group, the preoperative VAS score was 6.8 ± 0.94, compared to 7.4 ± 0.50 in the non-relief group, with no statistical difference observed in preoperative VAS (*P* > .05, Table 2). The postoperative VAS was 1.3 ± 0.87 in the relief group, in contrast to 7.3 ± 0.49 in the non-relief group. The mean VAS improvement rate was 1.06% ± 3.53% in the non-relief group, whereas it was 81.35% ± 13.20% in the relief group. Postoperative VAS were significantly lower in the relief group compared to the non-relief group, with a significantly higher VAS improvement rate (both *P* < .05, Table 2). An analysis of the entire cohort of patients who underwent discography from January 2019 to December 2024 revealed an incidence rate of 22.45% for the LPRAD phenomenon. Furthermore, no significant differences were identified in other outcomes between the 2 groups (*P* > .05, Table 2). In the relief group at the 3-month postoperative assessment for lower limb pain, 12 patients scored 0–2 on the VAS, 8 scored 3–5, and 2 scored 6–8. Over 50% of the relief group achieved medication-free pain resolution (VAS ≤ 2) that persisted throughout the 3 postoperative months. Nonoperative management proved effective in 8 patients scored 3–5 through oral analgesia monotherapy, whereas 2 patients scored 6–8 progressed to endoscopic surgery due to refractory symptoms (Fig. 2).

**Table 2:** Comparison of radiologic and operative outcomes between the two groups.

	Non-relief (n = 47)	Relief (n = 22)	*P* value
Preoperative VAS	7.4 (0.50)	6.8 (0.94)	NS
Postoperative VAS	7.3 (0.49)	1.3 (0.87)	<.05
VAS improvement rate	1.06% (3.53%)	81.35% (13.20%)	<.05
CT scan timing, min	123.81 (80.27)	102.32 (64.69)	NS
Positive discography			NS
None	23	10	
Low back	9	4	
Leg	13	4	
Both	2	4	
DDD			NS
1	0	1	
2	3	0	
3	1	2	
4	27	12	
5	16	7	

DDD = Dallas discogram description, NS = not significant, ROM = extension–flexion, VAS = visual analog scale.

## 4. Discussion

Since its initial documentation in 1948, discography has evolved into an invasive diagnostic procedure with established clinical significance for evaluating degenerative lumbar disc diseases.^[11–13]^ As understanding of the pathophysiology of degenerative lumbar intervertebral disc diseases progresses, discography has increasingly become a vital diagnostic tool for identifying the source of low back pain symptoms.^[14,15]^ This technique, guided by X-ray fluoroscopy or CT imaging, involves the injection of a contrast agent into the nucleus pulposus of the intervertebral disc, allowing for the assessment of the nucleus pulposus morphology and the replication of pain events. This process enables the evaluation of degenerative characteristics of the intervertebral disc and the identification of the affected segment in cases of multilevel lumbar disc herniation.^[16–19]^ The current study of intervertebral disc puncture therapy focuses on particular attention to therapeutic intervertebral disc block methodologies. Traditional approaches often utilize a combination of local anesthetics and corticosteroids for nerve root block, which can suppress inflammatory responses and alleviate pain.^[20]^ Biotherapeutics, such as platelet-rich plasma and peripheral blood mononuclear cells derived from human sources, are gaining prominence in research due to their potential to modulate inflammatory balance and exert therapeutic effects.^[21]^ However, the long-term efficacy, optimal administration protocols, and molecular mechanisms of these innovative therapies remain insufficiently understood, particularly regarding the survival and functional preservation of bioactive components within the hypoxic and nutrient-deficient microenvironment of intervertebral discs. This underscores the need for further investigation. Despite the significant role of discography in clinical diagnosis, there exists a substantial research gap concerning the analgesic effects following purely diagnostic discography, warranting urgent exploration.

Through an extensive literature review, we found no independent reports or studies examining the potential of discography to relieve radicular symptoms or its associated influencing factors in either English or Chinese databases. We identified significant differences in clinical outcomes between the relief group and non-relief group. Our study identified significant differences in clinical outcomes between the relief and non-relief groups following the application of discography. Postoperative VAS were significantly lower than those in the non-relief group, with a significantly higher VAS improvement rate (both *P* < .05, Table 2). The extent of pain reduction in the relief group was comparable to outcomes typically seen after endoscopic surgery, suggesting clinically meaningful symptom improvement. Moreover, no statistically significant change in VAS scores was detected before and after discography in the non-relief group (*P* > .05). This dichotomous response suggests that pain relief following discography may follow an “all-or-none” pattern rather than a continuous spectrum of efficacy. Specifically, patients either experienced substantial symptom relief or none at all, with no intermediate therapeutic response observed. Additionally, among the various parameters of disc herniation, only the superior–inferior diameter of the lumbar intervertebral disc protrusion showed a significant difference between the 2 groups. The relief group exhibited a reduced superior–inferior diameter of the lumbar intervertebral disc protrusion, suggesting that this parameter may be associated with the patient’s pain symptoms and surgical recovery. Furthermore, the administration of contrast agents can potentially impact the structural integrity of the intervertebral disc, leading to alterations in the morphology of the annulus fibrosus or nucleus pulposus. Such alterations may enable the compressive material to bypass previously compressed nerve roots, thereby alleviating the patient’s pain. A smaller herniation size may facilitate such changes, thereby increasing the probability of symptomatic relief.

In patients with Dallas discogram description type 5, where contrast agent leakage affects the spinal canal, the leaked agent exerts pressure on the compressed nerve root, resulting in its displacement. This displacement may reduce compression and subsequently diminish pain. A decreased superior–inferior diameter of the herniated disc may suggest a lower degree of nerve root compression, thereby increasing the likelihood of morphological changes induced by the contrast agent. Consequently, patients are more likely to experience pain relief following discography. In addition, the relief group exhibited better-preserved disc height, greater ROM, less severe disc degeneration, and lower intradiscal pressure. These factors may collectively contribute to a more responsive disc mechanical environment, where injected contrast can induce rapid pressure changes and structural adjustments in the annulus or nucleus, potentially alleviating nerve root irritation.

In our study, post-discography CT imaging in the relief group showed no marked reduction in herniation volume or significant nerve root displacement. This suggests that mechanical compression alone may not fully explain the pain relief observed. The existing body of literature suggests that lower limb pain associated with LDH is primarily attributed to 2 mechanisms: mechanical compression of nerve roots and inflammatory stimulation of these roots.^[22]^ The circulatory system of lumbar nerve roots may be compromised by herniated disc material, resulting in localized functional ischemia, inflammatory edema, and the accumulation of acidic metabolites, all of which contribute to lumbosacral radicular pain. Importantly, radicular pain can manifest even in the absence of significant mechanical compression, highlighting the pivotal role of inflammatory processes. In cases of LDH, compressed nerve roots consistently display varying degrees of inflammation.^[23,24]^ Pro-inflammatory mediators, such as tumor necrosis factor-alpha, interleukin-6, and interleukin-8, are released from degenerated discs and directly activate nociceptive pathways, thereby intensifying pain signals.^[25–30]^ In addition to mechanical compression, our study hypothesize that the dilution of inflammatory mediators may serve as a potential mechanism for symptom relief following discography. Assuming an average compressed nerve root diameter of 0.69 mm and a craniocaudal compressive lesion length of 5.7 mm, the calculated volume of the affected nerve root segment is 0.031 cm^3^. If 2 mL of contrast agent is administered, even a 1% leakage (0.02 mL) into the perineural space would correspond to 9.4 times the volume of the nerve root, significantly diluting local inflammatory mediators. Degenerated discs are characterized by an acidic microenvironment (pH 5.5–6.8),^[31]^ which enhances the activity of inflammatory mediators. The neutral pH of contrast agents (Iohexol, nonionic) may partially neutralize this acidity, thereby inhibiting inflammatory pathways and reducing neurogenic pain.

This dual mechanism provides a novel framework for understanding post-discography pain relief. Subsequent research should aim to substantiate these hypotheses by directly measuring the concentrations of inflammatory cytokines and monitoring pH variations within the perineural microenvironment.

### 4.1. Study limitations

First, its retrospective and single-center design may introduce selection bias and limit the generalizability of our findings. Second, the relatively small sample size, particularly in the relief group, may have reduced the statistical power to detect other potentially significant differences, Third, the statistical analysis was limited to bivariate comparisons without adjustment for potential confounding variables. Future prospective, multicenter studies with larger cohorts and more sophisticated statistical models are warranted to validate our findings.

## 5. Conclusions

We identified a phenomenon termed LPRAD, which was observed in 22.4% of our cohort. The findings suggest that increased disc height, greater segmental ROM, and smaller superior–inferior diameter of lumbar intervertebral disc protrusions may contribute to the occurrence of LPRAD in patients. The underlying mechanism may involve both mechanical and chemical factors, though further investigation is necessary to confirm these pathways. Future multicenter studies with larger sample sizes are needed to verify these observations and explore the prognostic factors associated with post-discography pain relief.

## Author contributions

**Data curation:** Zhenyuan Lu, Zhongyang Fan, Lu Lin, Yu Du.

**Resources:** Xiaolin Chen, Zhenyong Ke.

**Visualization:** Yang Wang, Yun Cheng.

**Writing – original draft:** Zhenyuan Lu.

**Writing – review & editing:** Xiaolin Chen, Zhenyong Ke.
